# The Pen and Plow

**Published:** 1875-05

**Authors:** 


*The Pen and Plow* is the title of a very well conducted and instructive monthly paper, published in New York, under the editorship of J. Payne Low, and devoted to agriculture, horticulture, landscape gardening and kindred subjects, and original and well selected matter besides.

It was formerly designated the "Farm and Fireside Journal. and is a welcome visitor wherever it is received.

